# Creating Respectful Workplaces for Nurses in Regional Acute Care Settings: Protocol for a Sequential Explanatory Mixed Methods Study

**DOI:** 10.2196/18643

**Published:** 2021-01-11

**Authors:** Natasha Hawkins, Sarah Jeong, Tony Smith

**Affiliations:** 1 The School of Nursing & Midwifery The University of Newcastle Australia Taree Australia; 2 The School of Nursing & Midwifery The University of Newcastle Australia Ourimbah Australia; 3 The University of Newcastle Department of Rural Health Taree Australia

**Keywords:** bullying, culture, negative behavior, nurses, methods, workforce

## Abstract

**Background:**

Negative workplace behaviour among nurses is an internationally recognised problem, despite the plethora of literature spanning several decades. The various forms of mistreatments and uncaring attitudes experienced by nurses include workplace aggression, incivility, bullying, harassment and horizontal violence. Negative behaviour has detrimental effects on the individual nurse, the organisation, the nursing profession and patients. Multi-level organisational interventions are warranted to influence the “civility norms” of the nursing profession.

**Objective:**

The aim of this study is to investigate the self-reported exposure to and experiences of negative workplace behaviours of nursing staff and their ways of coping in regional acute care hospitals in one Local Health District (LHD) in NSW before and after Respectful Workplace Workshops have been implemented within the organisation.

**Methods:**

This study employs a mixed methods sequential explanatory design with an embedded experimental component, underpinned by Social World’s Theory. This study will be carried out in four acute care regional hospitals from a Local Health District (LHD) in New South Wales (NSW), Australia. The nurse unit managers, registered nurses and new graduate nurses from the medical and surgical wards of all four hospitals will be invited to complete a pre-survey examining their experiences, perceptions and responses to negative workplace behaviour, and their ways of coping when exposed. Face-to-face educational workshops will then be implemented by the organisation at two of the four hospitals. The workshops are designed to increase awareness of negative workplace behaviour, the pathways to seek assistance and aims to create respectful workplaces. Commencing 3 months after completion of the workshop implementation, follow up surveys and interviews will then be undertaken at all four hospitals.

**Results:**

The findings from this research will enhance understanding of negative workplace behaviour occurring within the nursing social world and assess the effectiveness of the LHD’s Respectful Workplace Workshops upon the levels of negative workplace behaviour occurring. By integrating qualitative and quantitative findings it will allow for a dual perspective of the social world of nurses where negative and/or respectful workplace behaviours occur, and provide data grounded in individuals lived experiences, positioned in a macro context

**Conclusions:**

It is expected that evidence from this study will inform nursing practice, and future policy development aimed at creating respectful workplaces.

**Trial Registration:**

Australian New Zealand Clinical Trials Registry (Registration No. ACTRN12618002007213; 14 December 2018).

**International Registered Report Identifier (IRRID):**

PRR1-10.2196/18643

## Introduction

### Background

Various forms of negative workplace behavior have been reported across the health care professions, including in nursing [1,2], allied health professions [3], and medicine [4]. In addition to making working life unpleasant and stressful, the existence of a negative workplace culture risks patient safety [4]. The term negative workplace behavior is a euphemism that encompasses a wide range of undesirable behaviors, such as bullying (either physical or otherwise), harassment, horizontal violence, and incivility. The commonality is that the behaviors occur in the workplace; however, there are differences in definitions [2], as does the identity of the perpetrators. Bullying is characterized by repetitive acts that are directed at a person or group by one or more perpetrators in a position of power [5], whereas horizontal violence occurs between peers in equal positions [6]. In addition to these reported high-intensity behaviors, it has been suggested that incivility, as defined by Pearson et al [7] as “low-intensity deviant behavior with ambiguous intent to harm the target, in violation of workplace norms,” occurs daily and is accepted as a normal part of the nursing *socialization process*. Owing to the varying terms used in the literature, the term *negative workplace behavior* will be used throughout this paper to be inclusive of bullying, harassment, horizontal violence, and incivility.

Negative workplace behavior in nursing has been extensively explored, and there is a great variation in the reported incidences [1,2]. Regardless of this variation, it is widely accepted that negative workplace behavior continues to be a significant issue impacting nurses both personally and professionally [8,9] not only in Australia but internationally [2]. However, caution is necessary while comparing the reported prevalence rates of negative workplace behavior between countries due to differences in terminology, the lack of definitional consensus, and the multiplicity of assessment measures used in different studies. Reporting is also influenced by pre-existing intercultural differences in the tolerance and perceptions of bullying or similar behaviors [2,10].

Although negative workplace behavior entails the actions of an individual or a group, organizational culture, workloads, and leadership styles have all been shown to affect its occurrence [10]. It has been suggested that organizations seeking to mitigate negative workplace behaviors should aim to improve organizational processes that inform the management of bullying, implement skills-based training in communication and conflict management, and aim to promote accountability, transparency, and respectful peer alliances [11,12]. In the literature, there is little evidence of effective strategies that have been implemented in acute care settings to address negative workplace behavior experienced by nurses [2,13]. Of 18 previous quantitative studies that were recently reviewed critically, as reported elsewhere [2], all were descriptive and exploratory, as opposed to interventional studies. The available literature sanctions intervention at multiple levels to influence the *civility norms* of the nursing profession, clearly emphasizing on the modification of culture and practice [13]. It is evident in nursing as well as other disciplines that interventions with integrated, organization-wide approaches improve *civility* [13-16], whereas interventions focused on individuals have less impact [17,18]. Therefore, rather than focusing on individual interventions, such as targeting new graduate nurses (NGNs) to improve resilience [19], the research methodology described in this paper takes a multi-level approach. The protocol targets NGNs, registered nurses (RNs), and senior RNs in managerial positions. The study will investigate participants’ experiences of negative workplace behavior and their ways of coping when exposed in an environment where an intervention was being implemented at an organizational level concurrently, at the time the research was being undertaken.

### Theoretical Framework

The theoretical framework that underpins this research protocol is the Social Worlds Theory [20]. Social Worlds Theorists explain society as shaped and upheld via repeated interactions between individuals [21] and that society as a whole can be conceptualized as consisting of a mosaic of social worlds that both touch and intersect [22]. These social worlds refer to groups where there is *a set of common or joint activities or concerns, bound together by a network of communication* [23]. Social worlds develop around one primary activity (eg, delivery of patient care). There are also sites where these activities occur (eg, within the hospital setting) and technology relating to performing the activities is always involved (eg, clinical and technical skills). Each individual within the social world is engaged in a relevant activity; however, some individuals are, or believe themselves to be, more *authentically* belonging and more suited to that world [24]. Claims of authenticity can lead to conflict and power struggles and consequently to the creation of excommunicated individuals of social subworlds [24]. The ongoing conflict and the creation of subworlds within the primary social world of nursing is evident, and tension and conflict manifest in the form of negative workplace behavior.

## Methods

This study is ongoing and has been registered with the Australian New Zealand Clinical Trials Registry (Registration No. ACTRN12618002007213; December 14, 2018).

### Study Aims

The aim of this study is to investigate the self-reported exposure to and experiences of negative workplace behaviors of nursing staff and their ways of coping in regional acute care hospitals in one local health district (LHD) in New South Wales before and after Respectful Workplace Workshops have been implemented within the organization.

### The Study Design

This study uses a mixed methods, sequential explanatory design [25] with an embedded experimental component [25,26]. Data collection will occur in 3 distinct strands:

- Strand 1: an initial survey before the delivery of the Respectful Workplace Workshops by the organization (quantitative).
- Strand 2: follow-up surveys after conducting the workshops at 2 of the 4 hospitals involved in the study (quantitative).
- Strand 3: interviews with nursing staff from across all the hospitals involved in the study (qualitative).

**Figure 1:**
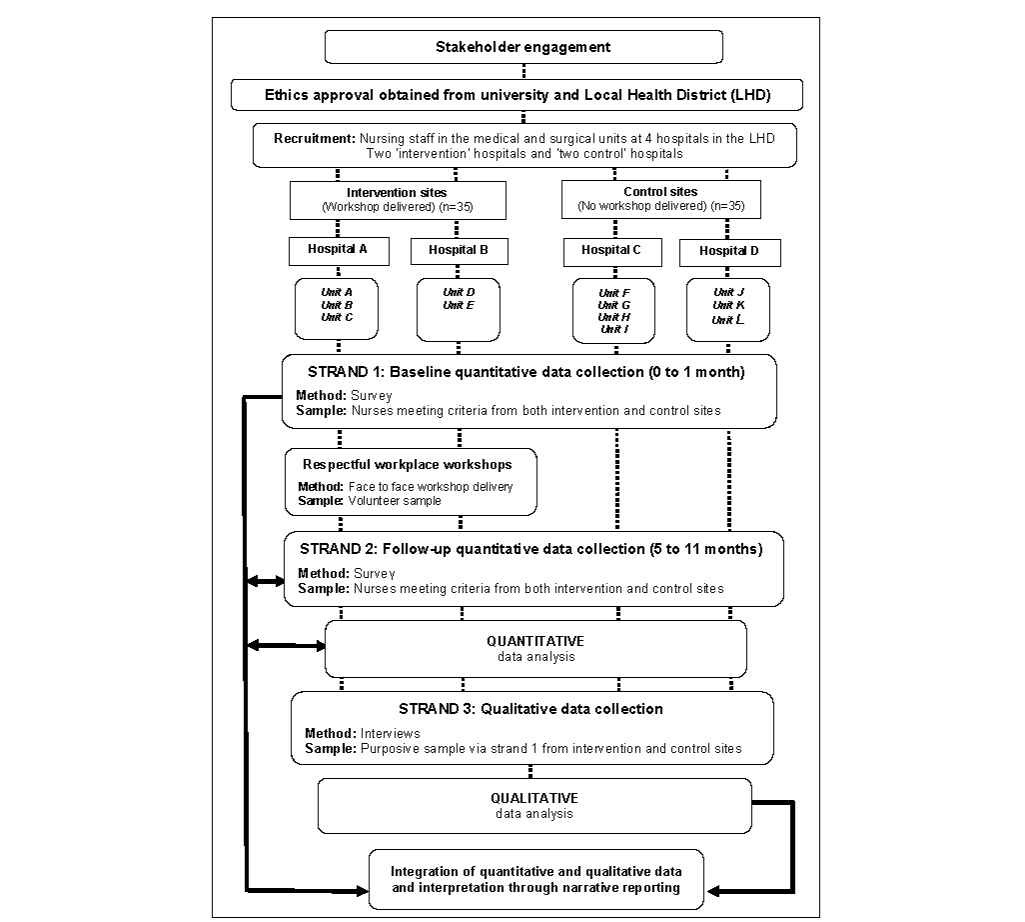
Flowchart showing the entire research process for the mixed methods study.

The sequence of the strands is presented in Figure 1. The mixed methods approach recognizes that neither quantitative nor qualitative study designs alone are able to capture the nuances of particular phenomena, whereas the combination of both takes advantage of their respective strengths [25]. Integrating qualitative and quantitative findings will allow triangulation and a broader perspective of the participants’ social worlds, with data grounded in individuals’ lived experiences, positioned in a macro-organizational context [27].

### Stakeholder Engagement

The research team collaborated early in the study design process with stakeholders from within the organization. Consultation took place with the LHD Respectful Workplace Workshop delivery team, the research committee, the executive director of nursing, and the directors of nursing, nurse unit managers (NUMS), and clinical nurse educators (CNEs) at each proposed hospital site. Stakeholder engagement in health care research is advocated to increase collaboration between researchers and users, thereby increasing research impact and knowledge translation [28]. This collaborative design process allowed for the negotiation of included hospitals to meet both researcher and organizational needs by assisting and aligning with the organization’s plan to deliver Respectful Workplace Workshops at the proposed sites. The consultation also allowed the identification of potential barriers in undertaking the study, including teaching workloads for the workshop delivery team and clinical workload and time required away from the wards for staff to attend the workshops.

### The Respectful Workplace Workshops

The Respectful Workplace Workshops will occur in the intervening period of data collection between strand 1 and strand 2. The workshops comprise 3 copyrighted face-to-face training modules developed for LHD delivery. The aim of the modules is to promote respectful workplace behavior by improving communication between staff members with the aim to recognize, manage, and mitigate negative workplace behavior [29]. These workshops have previously been delivered at other metropolitan hospitals within the LHD but not at those selected for this study.

The Respectful Workplace Team, an education division within the LHD, delivers the workshops, with all workshop facilitators having completed relevant prior training. The workshops require staff to attend face-to-face teaching sessions. Modules 1 and 2 require 2 hours of contact time and, to assist with rostering, the team has combined modules 1 and 2 into a single 4-hour workshop. Module 3 is only for nurse unit managers and requires attendance for a further 4 hours of face-to-face contact. The workshops will be made available to staff on various days over a 3-month period to increase attendance and minimize disruption.

The modules use a combination of training methods, including role-play, brainstorming, didactic teaching with PowerPoint presentations, and workbooks. The first two modules aim to challenge participants and encourage reflection on responsibilities and contributions to support a respectful workplace. These modules provide participants with a structured conversation template to assist with clear, direct, and respectful communication, allowing for role-playing of these conversations to assist the translation of theory into practice. The third module explores the manager’s role in supporting a respectful workplace and aims to improve managerial skills by using resolution pathways and coaching of other staff. Although workshop attendance is not compulsory, the directors or nursing have agreed to support and encourage attendance and roster staff accordingly.

### Setting, Sampling, and Participants

Study setting, sampling, and participant recruitment will be based on the inclusion criteria pertinent to the research aims [30]. To minimize selection and participant bias, comprehensive and rigorous criteria have been developed. Potential hospitals and units for inclusion were selected in consultation with the Respectful Workplace Team, the members of which are employed in the LHD to deliver Respectful Workplace Workshops in the hospitals. The selection of hospitals and units was based on the presence of minimal or no previous attendance by nurses at the Respectful Workplace Workshops. A total of 9 employees across the 4 hospitals were identified as having potentially attended the workshops previously at other hospitals within the health district. The hospitals chosen are of similar size, with similar service availability and case mix. Regional hospitals were selected because the phenomenon under investigation is of significant concern in nonmetropolitan locations, where ongoing staffing and recruitment issues exist [31]. Being in the same LHD, all 4 hospitals have the same centralized executive leadership, are subject to the same bullying and negative workplace behavior policies, and are subject to the same human research ethics committee governance processes. In total, 2 of the 4 hospitals were assigned to have the Respectful Workplace Workshops delivered within the 6-month study period; these hospitals (A and B) are referred to as the *intervention sites* in Figure 1. The other 2 hospitals did not have the workshop delivered until after data collection of strands 2 and 3 was completed. Those hospitals (C and D) are referred to as the *control sites* in Figure 1.

This study included 12 wards or units across 4 nonmetropolitan, regional acute care hospitals within the same New South Wales LHD. The units were selected on the basis that they were general medical or surgical wards, where most NGNs were employed.

Targeted study participants may be categorized as follows:

- NGNs in their first 12 months of practice following the completion of a Bachelor of Nursing degree.
- RNs who had been employed for more than 1 year at a minimum of 0.6 full-time equivalent.
- Senior RNs are employed in permanent leadership roles, who have managerial responsibilities for other staff members and patients, including NUMs, CNEs, and clinical nurse specialists.

Nursing staff known to have attended the workshops previously will be excluded from selection and others who indicate in the initial survey screening questions that they had previously attended the workshop will also be excluded from the data analysis. Owing to the relatively low number of previously exposed participants spread across 12 wards or units, the risk of contamination bias at a site level was considered minimal.

The participants will be allocated into clusters according to hospitals at which they are working; thus, they will be employed at hospitals where the workshops were either delivered or not delivered. Nested sampling will be undertaken for the qualitative and quantitative components of the study. The sample of the qualitative component is a subset of those who participated in the quantitative components. Survey participants will be a volunteer sample of nursing staff working in the medical and surgical wards in all 4 participating hospitals. The sample for the qualitative component of the study will be purposively chosen from survey respondents who will volunteer to be interviewed to represent the roles of the nurses and sites where they are working.

The total target population is 230 at the time of writing this paper, which includes 64 NGNs, 154 other RNs, and 12 senior RNs with managerial roles. Assuming a 30% response rate (n=69), 5% type 1 error, and 80% power to detect an effect size equivalent to 0.7 of the SD, it is anticipated that it will be necessary to recruit 35 participants from the hospitals where the Respectful Workplace Workshops will be delivered and 35 from those where no workshops are conducted.

### Strand 1 and 2: Surveys (Quantitative)

Surveys will be administered on 2 occasions, separated by several months. First, a baseline survey will be conducted across all 4 participating hospitals within the first month after initiation of the study, with the second follow-up survey conducted between 5 and 11 months after initiation. In the intervening period, the Respectful Workplace Workshops will be conducted at 2 of the 4 hospitals.

This component of the study is designed to investigate NGNs’, RNs’, and senior RNs’ experiences and perceptions of negative workplace behaviors occurring in medical and surgical wards as well as whether there are any observable differences over the period of the study between the two sites where the workshops will be conducted as compared with the other two sites. The survey will also examine the ways of coping by respondents after being exposed to negative workplace behaviors. The initial survey will capture data from the NGNs within their first 3 months of employment, when they are reportedly most vulnerable and require most support [32]. Participants do not need to complete the strand 1 survey or attend the workshops to participate in the strand 2 survey.

With careful consideration of the content and layout, the questionnaire was informed by a literature search to identify the validated instruments. The recruitment package consists of multiple parts, as follows:

- A participant information statement detailing the nature of the study according to the ethics requirements
- Demographic and background questions
- Negative Acts Questionnaire—Revised (NAQ-R) [33]
- Purpose-designed self-assessment of exposure to bullying and incivility questions
- Purpose-designed questions informed by the learning outcomes of the workshops
- Management of bullying and incivility questions (NUMs only)
- Ways of Coping Questionnaire (WCQ) [34]
- A separate consent form to return for volunteering to participate in a subsequent interview

Permission was granted for the use of the NAQ-R [33], which was originally designed to measure exposure to bullying in the workplace. This instrument consists of 22 items measuring exposure to negative workplace behaviors, with response alternatives on a 5-point Likert-type scale, where the higher the score, the greater the frequency of exposure to negative acts. The Cronbach α for the NAQ-R is .95 for the total scale, meaning that it has a high level of consistency between the questionnaire items. The NAQ-R has 3 subscales: person-related bullying, work-related bullying, and physically intimidating bullying [33]. Two additional items will be added to the NAQ-R to assess the perceived degree to which patient care is compromised or obstructed and whether respondents felt that they have been isolated from supportive peers. The addition of these items was justified by evidence from a recent integrative review of relevant literature [2].

The purpose-designed self-assessment of exposure to bullying questions also uses a 5-point Likert-type scale response, where higher scores indicate increased exposure. Before answering this section, respondents are asked to consider the definition of *workplace bullying* from the New South Wales Ministry of Health [35]. Participants were also asked whether they had been exposed to incivility at work over the previous month, with response alternatives as follows: 1=no; 2=yes, but only rarely; 3=yes, now and then; 4=yes, several times per week; and 5=yes, almost daily. For this question, the definition of *workplace incivility* is “lower level, subtle forms of workplace mistreatment.” Examples include: *having your ideas or opinions dismissed*; *having derogatory or demeaning remarks made about your work*; *eye rolling*; *feeling belittled or humiliated*; *and being stared at*, *watched*, *or being excluded from social activities*.

Participants are asked to identify the designation of the *perpetrator* of the negative workplace behavior to which they had been exposed. Response options are as follows: manager, colleagues (other RNs), endorsed enrolled nurses, assistants in nursing, patients, doctors, students, and others. The turnover intentions of participants will be measured on a Likert scale (strongly disagree, disagree, unsure, agree, and strongly agree) in response to the statement that “the impacts of bullying and incivility in my workplace make me think about leaving my current position.” Likert scale responses are also used as a self-assessment measure of participants’ capabilities to respond to and manage bullying and incivility. Participants are asked about policy awareness and their perceived capacity to use resolution pathways, challenge disrespectful behavior, and know when to escalate and ask for assistance.

The WCQ is a 66-item instrument that is designed to examine coping processes in stressful encounters [34]. Revised in 1985, it is in the public domain and no special permission was required for its use [34]. Respondents are asked to indicate to what extent they use particular strategies to cope when exposed to negative workplace behavior using the following responses: 0=not used, 1=used somewhat, 2=used quite a bit, and 3=used a great deal. The WCQ consists of 8 subdomains, including 1 problem-focused scale, 6 emotion-focused scales, and an eighth scale containing both problem-focused and emotion-focused items [34]. The Cronbach α for each scale ranged from .61 to .79 [34].

The NGNs, RNs, and senior RNs will be invited to complete the surveys on either hardcopy or on the internet via *REDCap* (Hunter Medical Research Institute, 2020), which is a secure web-based application for building and managing web-based surveys and databases. Details of how to access the web-based survey will be displayed on information posters in the wards or units, with responses uploaded automatically to the database. The web-based option allows nurses to participate without the risk of being seen taking a hardcopy from the ward or unit, whereas the alternative hardcopy option considers limitations in access to the internet. Hardcopies can be returned anonymously by either depositing them into brightly colored, sealed boxes in the staff rooms or sending them directly to the research team in the self-addressed, reply paid envelopes. To increase the response rates, all potential respondents will receive an email reminder from the new graduate coordinator at their particular hospital at 2 and 4 weeks after the initial survey distribution.

### Strand 3: Interviews (Qualitative)

The qualitative research component adheres to the COREQ (Consolidated Criteria for Reporting Qualitative Studies) [36]. A completed COREQ checklist has been appended as Multimedia Appendix 1 to this study.

The qualitative strand occurring after preliminary quantitative data analysis aims to broaden and deepen the understanding of nurses’ experiences and perceptions of workplace behavior by allowing exploration through dialog in semistructured, one-on-one interviews [27]. To participate, informants will be purposively selected to be representative of nursing roles and hospital sites from the consenting participants in strand 1 (Figure 1). The informants need not attend the workshops to participate in an interview but must work on one of the wards included in the sampling frame. The interviews are expected to last up to 1 hour and will be audio-recorded, then transcribed verbatim by a transcription service with which the university has a confidentiality agreement for subsequent content analysis. The sample size in the qualitative strand cannot be postulated, as it will be determined by developing theoretical categories [37]. Data collection and analysis will occur concurrently, and it is anticipated that interviews will continue until such time that no *new theoretical insights nor new properties of core theoretical categories emerge* [37].

### Quantitative Data Analysis

The quantitative data will be analyzed using Stata 14 (TM; StataCorp LP), a statistical software that enables users to analyze, manage, and produce graphical presentations of data [38]. Participant characteristics will be summarized separately for intervention and control sites. Differences between groups for categorical variables will be assessed using chi-squared tests (or Fisher exact, the nonparametric equivalent) and Student's *t* tests for continuous variables. Differences between intervention and control sites in exposure to bullying (as assessed by the NAQ-R) and ways of coping (as assessed by the WCQ) will be assessed for all nurses at strand 2. Statistician support will be pursued to assist with analysis.

### Qualitative Data Analysis

The qualitative data will be organized using NVivo software (version 11; QSR International) [39]. The data analysis process will be guided by the Straussian Grounded Theory (SGT) [40,41], the method of choice for researchers framing their research within the Social Worlds Theory [42]. The aim of SGT is to generate an *explanatory theory that closely approximates the reality it represents* [43] and explore not only the phenomenon but also how the actors involved respond to this phenomenon and the consequences of their actions [40]. The SGT method includes a 3-stage approach: open coding, axial coding, and selective coding [40,44]. In the initial stage of open coding, the researcher is immersed in the transcribed interview data line-by-line using constant comparison to identify the categories of data [40,44]. Constant comparison involves reading and rereading transcripts, constantly comparing similarities and differences and sorting data into categories [45]. The axial coding stage involves the identification of relationships between data categories [40,44]. One of the key features of SGT is using a coding paradigm to assist with analyzing, refining, and aligning categories [40]. In the final, selective coding stage, categories are refined and integrated, ultimately leading to a small number of core categories that will link directly to the data [40,44]. The researchers will ensure the trustworthiness of the study by considering the validity standards that ensure rigor, confirmability, credibility, dependability, and transferability [46].

### Data Integration

Owing to the separative approach to data analysis, where quantitative and qualitative data are collected and analyzed separately [47,48], the integration of data will occur in the interpretation phase to assimilate the 2 analyses of the phenomena [47-50]. This approach is known as meta-inference and is achieved through a narrative reporting process, writing both qualitative and quantitative findings together on a theme-by-theme or concept-by-concept basis [51]. There are inherent challenges in undertaking data integration in mixed methods studies, and there are few examples of mixed methods research integration [52]. Challenges include researchers’ experience and methodological preferences; the nature of the data and avoidance of placing greater emphasis to one set of findings over the other; the design and timing of the phases of the study; and publication requirements and the target audience [52].

### Ethical Considerations

The research project adheres to the National Statement on Ethical Conduct in Human Research Council [53] and is approved by both the LHD and university ethics committees. Potential participants will be informed of the aim and requirements for participation via a participant information statement to ensure informed consent. Participants will be informed of their rights, privacy and confidentiality, the usage and storage of information, contact for complaints, and dissemination of results in the participant information statement. Completion of the survey will indicate respondent consent for the quantitative components of the study. A written consent form for an individual interview will be obtained before the interview. Given that the nature of the research topic may elicit an emotional response, participants can bring a support person with them to the interview, if they wish. The contact details of the free Employee Assistance Program counseling service will be made available as well as contact details for Beyond Blue [54], a national not-for-profit organization providing online and telephone support for individuals experiencing distress. Participants’ autonomy will be respected and they may withdraw from the study at any time without any adverse consequences. Data will be stored confidentially and will be deidentified.

## Results

This study is currently ongoing and the results, using the recruitment, data collection, and data analysis methodology described above, are expected to be available no later than the end of 2022.

## Discussion

### Principal Findings

This paper has presented the protocol of an ongoing study that aims to investigate the nurses’ experiences of negative workplace behavior and their ways of coping in an environment where an intervention is being implemented by the health service in which they are employed. The Respectful Workplace Workshops aim to empower nurses to recognize negative workplace behavior as well as promote and reinforce *civility* and *workplace courtesy* to create a respectful and supportive workplace, where nurses feel safe to practice. In addition to the before-and-after approach, the study design has included both *intervention* and *control* sites, making it possible to compare the results between the sites where the workshops are delivered and sites where the education has been temporarily withheld until after the research has been completed. It is anticipated that at the sites where the workshops occur, there will be an overall reduction in the levels of perceived negative workplace behaviors, as identified by the NAQ-R. Given that there have been limited strategies implemented in the health care setting to address negative workplace behaviors, this study may help understand to what extent, if at all, an intervention designed to intervene at professional and organizational levels is effective for the workplace culture and climate. Hence, the research relates more to organizational, macro-level *environmental* consequences of negative workplace behavior than on individuals, although the latter is also of considerable interest.

One of the benefits of undertaking this research in relatively small regions, as opposed to major metropolitan hospitals, is that the target population is more confined and therefore less subject to external influences that may create bias or confound results. Regional hospitals have a smaller number of staff members, with a more limited rotation of rosters, making the recruitment of study participants more targeted. Such hospitals and wards also tend to be more closely knit, with staff members who often know each other well and have worked together for long periods. Although a core group of long-term staff members may exist, it is also a reality that staff turnover is problematic in nonmetropolitan locations, where staff cannot be readily replaced should they decide to leave. It may be that the long-term staff members’ attitudes, opinions, and values affect the general tenor of the social world they inhabit, and it may be perceived that new staff do not belong. This can be exacerbated if the new recruits are younger and less experienced. It is expected that this study will explore such factors, especially in the qualitative component.

The study design and methodology have several strengths. First, by undertaking mixed methods research, it will provide different perspectives about the same phenomenon. This study will also use the inclusive term *negative workplace behaviors* to capture the conceptual differences and variety of behaviors. Unclear, definitional consensus and clarity of the concept of bullying, harassment, horizontal violence, and incivility within the nursing profession suggests that the previous research had lacked focus, direction, and depth [2,55]. A synthesis of terms and greater conceptual clarity may improve identification, support, and intervention at personal, professional, and organizational levels. This inclusivity has the potential to provide insights into the current level of various forms of negative workplace behaviors to which NGNs as well as more experienced RNs are exposed. To the best of our knowledge, this is the first such inclusive approach to investigate this issue within the social world of nursing practice.

### Potential Limitations

Though inclusive of 4 regional hospitals and 12 wards, the findings from this study will be limited to a single LHD with one overarching management team, which affects generalizability. In addition, there are a number of threats to internal and external validity [56]. Care is necessary in the selection of the *control* and *intervention* groups to ensure they are of equal size and comparable, so as to minimize confounding variables [55]. Maturation also needs to be considered [56], including the reflection of natural changes within the participants over the duration of the study. For example, a new graduate’s skills may improve, so that they are increasingly *fitting in* and *getting the job done* and perhaps less subject to negative behaviors. The research team also needs to consider that an unplanned event may occur during the study, which may impact the results unintentionally, such as staff changes or change of manager [56]. Low survey response rates and difficulty recruiting for interviews may also be a potential limitation. Owing to the sensitive nature of the research topic, staff members may be reluctant to participate. Attendance at the Respectful Workplace Workshops is also reliant upon managerial engagement to maximize attendance from each ward.

### Conclusions

The findings from this research will add to the volume of literature on negative workplace behavior in the health care professions; however, it will add new perspectives through using multiple, previously validated survey instruments in combination with qualitative, in-depth interviews. The integration of quantitative and qualitative methods will allow for a dual perspective contextualized with the theoretical lens of the Social Worlds Theory to provide data grounded in individuals’ lived experiences and positioned in a macro-organizational reality. A further perspective is provided by the opportunity to perform the research in conjunction with the delivery of the LHD’s Respectful Workplace Workshops. By collecting pre- and postworkshop data and from sites both exposed and not exposed to the workshops, it is expected to add insights into the efficacy of such interventions. It is anticipated that evidence from this study will inform future nursing practice and policy development aimed at creating respectful workplaces.

## Appendix

Multimedia Appendix 1 Consolidated criteria for reporting qualitative (COREQ) checklist.

## Abbreviations

CNE: clinical nurse educator
COREQ: consolidated criteria for reporting qualitative
LHD: local health district
NAQ-R: Negative Acts Questionnaire–Revised
NUM: nurse unit manager
RN: registered nurse
SGT: Straussian Grounded Theory
WCQ: ways of coping questionnaire
