# Supplementary material for: Creating Respectful Workplaces for Nurses in Regional Acute Care Settings: Protocol for a Sequential Explanatory Mixed Methods Study
Source: JMIR Res Protoc. 2021 Jan 11;10(1):e18643. doi: 10.2196/18643 (PMC7834930; doi:10.2196/18643)
Supplement: Multimedia Appendix 1 [file resprot_v10i1e18643_app1.docx]

**Table 1: COREQ (COnsolidated criteria for REporting Qualitative research) Checklist**

| **Topic** | **Guide Questions/Description** |
| --- | --- |
| **Interviewer/facilitator** | All interviews will be conducted by the same member of the study team |
| **Credentials** | The interviewer will be a PhD candidate |
| **Occupation** | The interviewer will be employed as a part time Nurse Academic |
| **Gender** | The interviewer will be female |
| **Experience and training** | The interviewer will have successfully completed the qualitative subject component of her PhD studies and will conduct a pilot interview under the guidance of a PhD supervisor. |
| **Relationship established** | Due to the interviewer having worked previously as a clinician, there is the possibility that a minority of the participants from one site will have a previously established relationship with the interviewer (colleagues). The interviewer will be transparent by identifying herself as a researcher and the interviewer with great interest in interviewees’ experiences in the research topic. |
| **Participant knowledge of the interviewer** | Participants will be advised of the researchers goals, study aims, requirements and reasons for undertaking the research via the standardised participant information statement. |
| **Interviewer characteristics** | The researcher has lived experience of the research topic which led to her pre-existing interest in the research topic. Participants will be informed of the reasons for undertaking the research via the standardised participant information statement and this will also be discussed at the beginning of each interview. Participants will be aware that the interviewer is a registered nurse and that the research is being undertaken as part of her PhD thesis. |
| **Methodological orientation and Theory** | The qualitative component of this study will be underpinned by Social Worlds Theory and analysed using a Straussian version of Grounded Theory. |
| **Sampling** | Participants will be asked to volunteer, and from that volunteer sample Potential interviewees will be purposively sampled to be representative of nursing roles and sites (intervention and control). |
| **Method of approach** | Initially potential participants will be informed of the study in a face to face information session, and information packs including consent for interview will be left in the tea rooms of selected wards. After the returning of consent forms, those who have volunteered will be purposively sampled and contacted via email to schedule the interview. |
| **Sample size** | N = 230 |
| **Non-participation** | Due to the sensitive nature of the research topic it is expected that some participants will not feel comfortable participating in the interviews, however they will still be able to complete the survey and/or the educational intervention (intervention sites only). |
| **Setting of data collection** | Data will be collected via face to face interviews at a mutually convenient time in private setting. |
| **Presence of non- participants** | Due to the sensitive nature of the topic, participants will be given the option to bring along a support person. |
| **Description of sample** | The sample will include Nurse unit managers, Registered nurses, Clinical nurse educators, Clinical nurse specialists and new graduate nurses from regional acute care settings. |
| **Interview guide** | The research team will develop the semi-structured interview guide, which will then be pilot tested and further refined prior to interviews. |
| **Repeat interviews** | At this stage there is no plan to repeat interviews, however if further clarification or deeper insight is required, it will be sought via email (due to geographical locations) from the participants. |
| **Audio/visual recording** | The research team intend to audio record the interviews with the participants consent. |
| **Field notes** | The interviewer will make field notes after each interview. |
| **Duration** | It is anticipated that each interview will take approx. 45 mins. |
| **Topic** | **Guide Questions/Description** |
| **Data saturation** | Analysis and data collection will occur concurrently and Interviews will continue until such time that no ‘new theoretical insights nor new properties of core theoretical categories emerge’ |
| **Transcripts returned** | The research team intend on sending interview transcripts back to participants for confirmation of validity. |
| **Number of data coders** | All three members of the research team will be involved in open coding a sample of transcripts. |
| **Description of the coding tree** | The involvement of all three members in the coding process will allow for comparison and meaningful discussions about how the coding will be approached and to develop the codebook. Coding discrepancies will be resolved through discussion and the list of codes will be refined until consensus is reached. An audit trail be established showing the analysis process from raw data in the transcripts through to final selective coding and the emersion of themes. |
| **Derivation of themes** | Themes will be developed in stage 2 (axial coding) and stage 3 (selective coding) and involve all three members of the research team. |
| **Software** | The research team intend to use NVIVO qualitative software |
| **Participant checking** | A summary of key findings will be shared with participants following data analysis to ensure authenticity and validity. |
| **Quotations presented** | Quotations from multiple participants will be used to present the findings to ensure transparency and trustworthiness. |
| **Data and ﬁndings consistent** | The research team intend on including an audit trail to ensure transparency of the data analysis, and demonstrate association to the reported findings. |
| **Clarity of major themes** | It is intended to use headings and sub-headings to promote clarity when presenting the findings. |
| **Clarity of minor themes** | Minor themes and description of diverse cases will also be included in the findings where applicable. |
| Developed from: Tong A, Sainsbury P, Craig J. Consolidated criteria for reporting qualitative research (COREQ): a 32-item checklist for interviews and focus groups. *International Journal for Quality in Health Care*. 2007. Volume 19, Number 6: pp. 349 – 357 | |
